# ZnMn_2_O_4_/V_2_CT_x_ Composites Prepared as an Anode Material via High-Temperature Calcination Method for Optimized Li-Ion Batteries

**DOI:** 10.3390/mi15070828

**Published:** 2024-06-27

**Authors:** Ji Li, Yu Wang, Xinyuan Pei, Chunhe Zhou, Qing Zhao, Ming Lu, Wenjuan Han, Li Wang

**Affiliations:** 1 Key Laboratory of Functional Materials Physics and Chemistry of the Ministry of Education, Jilin Normal University, Changchun 130103, China; liji@jlnu.edu.cn (J.L.); peixyyy@163.com (X.P.); zhouch342@163.com (C.Z.); zhaoqing19991229@126.com (Q.Z.); hanwj@jlnu.edu.cn (W.H.); 2 Joint Laboratory of MXene Materials, Jilin Normal University, Changchun 130103, China; 3 State Key Laboratory of Superhard Materials, College of Physics, Jilin University, Changchun 130012, China; yu_w20@mails.jlu.edu.cn

**Keywords:** ZnMn_2_O_4_, V_2_CT_x_ MXene, composite, lithium-ion batteries

## Abstract

The ZnMn_2_O_4_/V_2_CT_x_ composites with a lamellar rod-like bond structure were successfully synthesized through high-temperature calcination at 300 °C, aiming to enhance the Li storage properties of spinel-type ZnMn_2_O_4_ anode materials for lithium-ion batteries. Moreover, even though the electrode of the composites obtained at 300 °C had a nominal specific capacity of 100 mAh g^−1^, it exhibited an impressive specific discharge capacity of 163 mAh g^−1^ after undergoing 100 cycles. This represents an approximate increase of 64% compared to that observed in the pure ZnMn_2_O_4_ electrode (99.5 mAh g^−1^). The remarkable performance of the composite can be credited to the collaborative impact between ZnMn_2_O_4_ and V_2_CT_x_, leading to a substantial improvement in its lithium ion storage capacity. Therefore, this study offers valuable insights into developing cost-effective, safe, and easily prepared anode materials.

## 1. Introduction

Recently, human society has witnessed a remarkable surge in progress, which has been accompanied by two significant challenges in the energy and environmental sectors that present major obstacles to the formulation of a national economic strategy [1,2]. Lithium-ion batteries (LIBs) have become widely used in various industries due to their exceptional characteristics, including a high capacity for storing energy, a minimal loss of charge over time, a lightweight construction, and an environmentally friendly nature. They play an indispensable role as vital energy storage devices that drive technological advancements and foster sustainable development [3,4,5]. Although LIBs exhibit exceptional performance in various aspects, they also possess certain drawbacks such as safety concerns, limited cycle life, temperature sensitivity, and capacity decay. Nevertheless, through continuous advancements in science and technology, along with technological enhancements, diligent efforts are being made to overcome these challenges. The ultimate goal is to further enhance the performance and safety of LIBs while promoting their extensive utilization across diverse fields [6,7,8].

Mixed transition metal oxides (MTMOs), exhibiting a spinel structure AB_2_O_4_, where A denotes divalent metal ions, such as Cu^2+^, Zn^2+^, Mn^2+^, and Mg^2+^, and B represents trivalent metal ions, including Fe^3+^, Al^3+^, Co^3+^, etc., have garnered significant attention owing to their remarkable electrochemical properties [9], being conversion anode materials exhibiting a theoretical capacity ranging from 700 to 1000 mAh g^−1^, involving the transfer of at least eight moles of electrons during the initial discharge process. Among the various MTMOs, ZnMn_2_O_4_ exhibits two distinct lithium intercalation mechanisms (conversion and alloying), allowing it to achieve an initial discharge treatment with a theoretical capacity of 1008 mAh g^−1^, surpassing other MTMOs without zinc [10,11]. In comparison to most Co- or Fe-based oxides, ZnMn_2_O_4_ operates within a voltage range of only 1.2–1.5 V (vs. Li^+^/Li), thereby expanding its potential window to battery applications [12]. Moreover, its abundant raw material sources, cost-effectiveness, and near-non-toxicity contribute to its suitability for large-scale utilization [13]. However, the inevitable volume changes during charge and discharge pose challenges in practical applications by causing structural collapse and comminution of the anode material, as well as reducing its capacity [14]. Additionally, similar to other MTMOs, ZnMn_2_O_4_ exhibits low electronic conductivity, which further hampers its performance. To address these limitations effectively, various strategies involve combining ZnMn_2_O_4_ with auxiliary materials, such as carbon nanotubes (CNTs) or graphene, that facilitate electron and ion transport while mitigating adverse effects associated with volumetric changes occurring throughout battery cycling [15,16].

The MXene material is a two-dimensional (2D) layered material characterized by its highly conductive core and exceptional dispersibility in aqueous solutions owing to its hydrophilic surface [17]. Its unique nature makes fleck-like 2D MXene commonly used for constructing high-level structures for LiBs [18,19]. In this study, V_2_CT_x_ MXene was utilized to enhance the low conductivity, slow ion diffusion, and volume expansion of ZnMn_2_O_4_. An effective method for synthesizing a ZnMn_2_O_4_/V_2_CT_x_ composite electrode is reported, aiming to (1) develop a simple and applicable approach for preparing composite materials and (2) characterize the correlation between the morphology, structure, and electrochemical characteristics of ZnMn_2_O_4_/V_2_CT_x_. Additionally, rational energy storage mechanisms have been proposed based on cyclic voltammetry tests. This work provides valuable data supporting the enhancement of the electrochemical properties of ZnMn_2_O_4_.

## 2. Experimental Methods

### 2.1. Materials

The V_2_AlC powder was supplied by 11 technology Co., Ltd. (China). Zinc sulfate heptahydrate (ZnSO_4_·7H_2_O), anhydrous oxalic acid (C_2_H_2_O_4_), and hydrochloric acid (HCl) were supplied by Shanghai Aladdin Biochemical Technology Co., Ltd. (China). Ammonium fluoride (NH_4_F) and polyethylene glycol 400 (H(OCH_2_CH_2_)_n_ OH) were supplied by Sinopharm Chemical Reagent Co., Ltd. (China). Manganese chloride (MnCl_2_·4H_2_O) was supplied by Tianjin Damao Chemical Reagent Factory. All chemicals and reagents utilized in the research are analyzed-grade or higher purity, obviating the need for addition purification.

### 2.2. Preparation Methods

#### 2.2.1. Preparation of ZnMn_2_O_4_ Micron Rods

The mixture of 20 mL anhydrous ethanol, 10 mL polyethylene glycol 400, and 10 mL deionized water was supplemented with 0.18 g of anhydrous oxalic acid (C_2_H_2_O_4_). Subsequently, the solution was further enriched with 0.144 g of zinc sulfate heptahydrate (ZnSO_4_·7H_2_O) and 0.198 g of manganese chloride (MnCl_2_·4H_2_O), followed by stirring at a temperature of 300 K for a period of 6 h. After being subjected to centrifugation at a speed of 5000 rpm for 5 min, the resulting precipitates underwent three washes with pure water and two washes with ethanol. Subsequently, they were dried in a preheated oven maintained at a temperature of 60 °C for up to 12 h to obtain precursor materials in the form of rods. The obtained precursor underwent calcination in a muffle furnace starting from room temperature until reaching a final temperature of 400 °C at an increment rate of 5 °C/min, followed by holding it steady for two hours prior to a natural cooling process, resulting in the formation of ZnMn_2_O_4_ micron rods labeled as the ZMO sample.

#### 2.2.2. Preparation of V_2_CT_x_ MXene by Hydrochloric Acid and Ammonium Fluoride Etching Method

The plastic beaker with a capacity of 50 mL was filled by combining 20 mL of hydrochloric acid and an equal volume of deionized water, and NH_4_F powder particles weighing 2.0 g were introduced into the mixture. The solution underwent magnetic stirring for a duration of 0.5 h at a temperature of 300 K. Subsequently, 2.0 g of V_2_AlC powder was gently added to the beaker and stirred for an additional 5 min. The mixture obtained was subsequently transferred into a 100 mL autoclave lined with Teflon and underwent hydrothermal treatment at a temperature of 120 °C for a period of 24 h. Afterwards, the remaining black sediment underwent several rinses with deionized water until the liquid above it had a pH value of ≥5. This was followed by an extra wash using alcohol. The damp black sediment produced from this procedure underwent filtration at a speed of 4500 rpm for 5 min, followed by dehydration in a vacuum oven set to 60 °C for a period of 12 h, resulting in the acquisition of the V_2_CT_x_ MXene specimen.

#### 2.2.3. Preparation of ZnMn_2_O_4_/V_2_CT_x_ Composite by High Temperature Calcination Method

In this method, 1.0 g ZnMn_2_O_4_ and 1.0 g V_2_CT_x_ MXene were ground together in a grinder until achieving homogeneity. The resulting mixture was divided into thirds: one portion remained uncalcined and labeled as the ZMO/V_2_CT_x_ sample; while the other two portions were calcined by air in the muffle furnace at heating rates of 5 °C/min, and the temperature reached 300 °C and 400 °C, respectively, and was kept for 2 h before natural cooling. The corresponding samples were designated as ZMO/V_2_CT_x_-300 and ZMO/V_2_CT_x_-400.

### 2.3. Characterization

The XRD patterns were acquired utilizing a Rigaku D/Max-2500 X-ray diffractometer (Japan) within the range of 5–65°. Raman spectroscopic investigations were conducted employing a Renishaw in Via RFS100/S micro-Raman spectrometer (Britain) with an excitation wavelength of 514 nm. Fourier transform infrared (FTIR) spectroscopy analysis was performed using a Thermo Scientific Nicolet iS50 spectrophotometer (USA). The surface morphology and composition analysis were carried out utilizing a JEOL JSM-7800F scanning electron microscope (SEM) (Japan). The surface composition was determined through X-ray photoelectron spectroscopy (XPS) using an Escalab 250Xi spectrometer (USA).

### 2.4. Electrochemical Tests

The electrochemical performance of the prepared samples was investigated employing the 2032 button battery technique, wherein a copper sheet was utilized as the substrate for coating the sample to create an anode, while lithium ions were employed as the reference electrode.

The active ingredient (sample obtained), conductive material (acetylene black), and binder (PVDF) were combined in a ratio of 8:1:1 by weight and dissolved in N-methyl pyrrolidone (NMP) solvent to create a slurry. This slurry was then applied onto circular copper sheets with a radius of 6 mm, followed by vacuum drying at 60 °C for 12 h. The electrochemical performance of the prepared electrodes was examined using cyclic voltammetry (CV) and constant current charge–discharge (GCD) measurements, conducted on electrochemical workstations (Chenhua CHI660E and Metrohm Autolab PGSTAT302N) (Switzerland), as well as a battery test system NEWARE CT-4008T (China). All electrochemical experiments utilized approximately 1 mg of active ingredients, and the mass loading of the active materials in the electrode was 1 mg/cm^2^.

## 3. Results and Discussion

### 3.1. Structural Characterization

**Figure 1:**
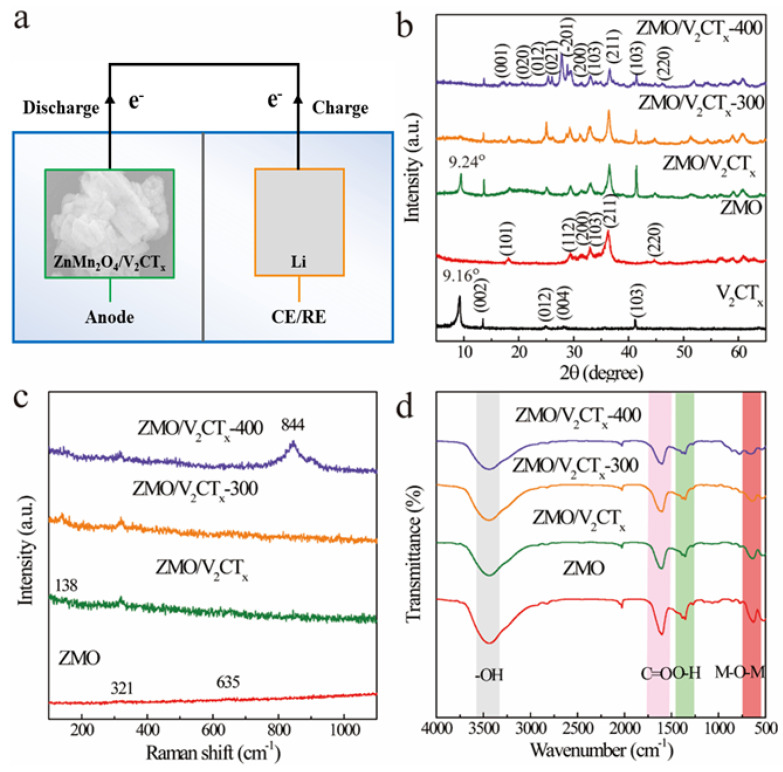
Battery configuration diagram of ZnMn_2_O_4_/V_2_CT_x_ (**a**); XRD pattern of V_2_CT_x_ MXene, ZMO, and ZnMn_2_O_4_/V_2_CT_x_ (**b**); Raman spectra of ZMO and ZnMn_2_O_4_/V_2_CT_x_ (**c**); FTIR spectra of ZMO and ZnMn_2_O_4_/V_2_CT_x_ (**d**).

In Figure 1a, an anode composed of ZnMn_2_O_4_/V_2_CT_x_ and a reference electrode consisting of a lithium sheet were utilized to fabricate LIBs. The two parallel electrodes were separated by a filter paper.

Figure 1b clearly illustrates the diffraction peaks of ZMO, which match well with standard PDF#71-2499. After etching V_2_AlC (PDF#29-0101) with NH_4_F+HCl, the characteristic diffraction peak at approximately 9.16° can be attributed to the (002) plane of V_2_CTx MXene. The results demonstrate successful fabrication of pure ZnMn_2_O4 and V_2_CT_x_ MXene materials. For the ZMO/V_2_CT_x_ sample, observed peaks correspond to crystal planes for both ZnMn_2_O4 and V_2_CT_x_ MXene without any significant impurity peak detected. It is noteworthy that compared to V_2_CT_x_ alone, in the ZMO/V_2_CT_x_ composite material, there is a slight shift in position from 9.16° to 9.24° for peak (002), indicating a more compact and stacked structure resulting from the grinding process on V_2_CT_x_ MXene. Upon calcination at 300 °C, the XRD pattern shows no apparent changes except for a gradual decrease in diffraction peak intensity of V_2_CT_x_ MXene, suggesting structural breakdown at high temperatures. However, when the temperature reaches 400 °C, the characteristic peaks strength of ZnMn_2_O_4_ gradually increases, along with the appearance of MnV_7_O_14_ phase (PDF#89-0484). As shown in Figure 1c, the Raman spectroscopy tests demonstrate that the 321 cm^−1^ Raman peak observed in the ZMO electrode is a result of the vibration of the Zn-O bond within the tetrahedral structure of ZnFe_2_O_4_. Additionally, it can be inferred from the presence of a 635 cm^−1^ Raman peak that there are symmetric stretching vibrations occurring in MnO_6_ groups, indicating the existence of a spinel phase. The peak at around 138 cm^−1^ represents a characteristic feature of V_2_CT_x_ MXene, confirming successful preparation of the ZnMn_2_O_4_/V_2_CT_x_ composite sample. Additionally, a characteristic peak near 844 cm^−1^ confirms the synthesis of Mn_2_V_2_O_7_ for ZMO/V_2_CT_x_-400 and is consistent with the XRD results. The FTIR spectra for synthesized ZMO and ZnMn_2_O_4_/V_2_CT_x_ are plotted over a range spanning from 400 to 4000 cm^−1^ and are shown in Figure 1d. In terms of pure ZMO (as seen in figure), an observed peak appears between regions ranging from 500 to 700 cm^−1^, which may be due to M-O-M (where M = Zn, Mn) bonding; weak bands at approximately 1001.84 cm^−1^ correspond to -OH vibrations; strong absorption around 1613 cm^−1^ arises from C-O stretching vibrations; and finally, the peak located at 3436 cm^−1^ corresponds to -OH stretching vibrations. The FTIR spectra for ZMO/V_2_CT_x_, ZMO/V_2_CT_x_-300, and ZMO/V_2_CT_x_-400 are generally consistent with those obtained for pure ZMO, except for the overall weaker intensity.

**Figure 2:**
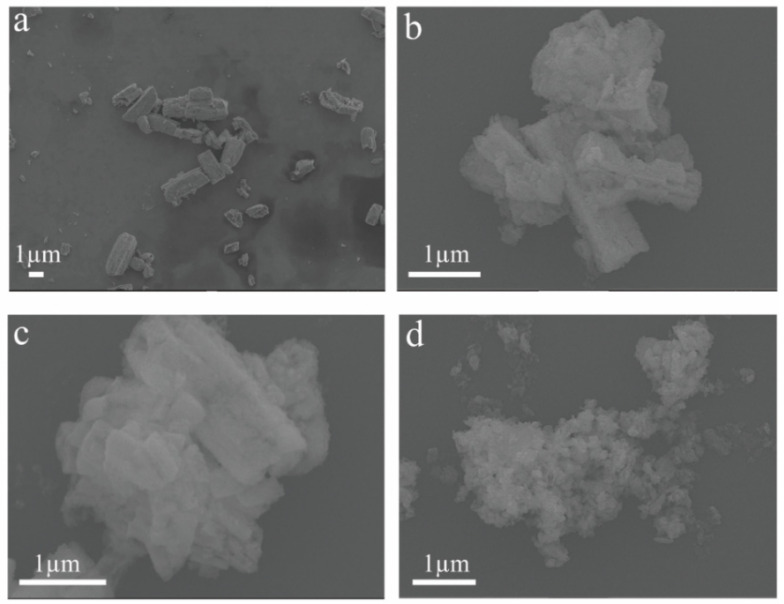
SEM images of ZMO (**a**); ZMO/V_2_CT_x_ (**b**); ZMO/V_2_CT_x_-300 (**c**); and ZMO/V_2_CT_x_-400 (**d**).

**Figure 3:**
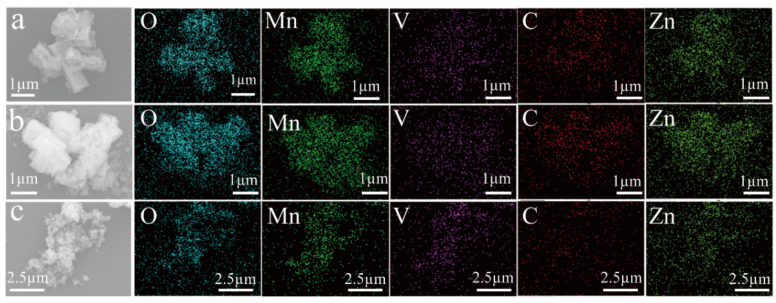
Elemental mappings of ZMO/V_2_CT_x_ (**a**); ZMO/V_2_CT_x_-300 (**b**); and ZMO/V_2_CT_x_-400 (**c**).

The morphology of ZMO and ZnMn_2_O_4_/V_2_CT_x_ composites is depicted in Figure 2, revealing the binding between the micrometer rods of ZMO and the lamellae of V_2_CT_x_ MXene. As the temperature increases, the bonding becomes more pronounced, leading to a spherical structure at 400 °C, with severe aggregation phenomena observed. The presence of Zn, Mn, O, V, and C elements on the sample surface is confirmed through elemental mapping (Figure 3).

**Figure 4:**
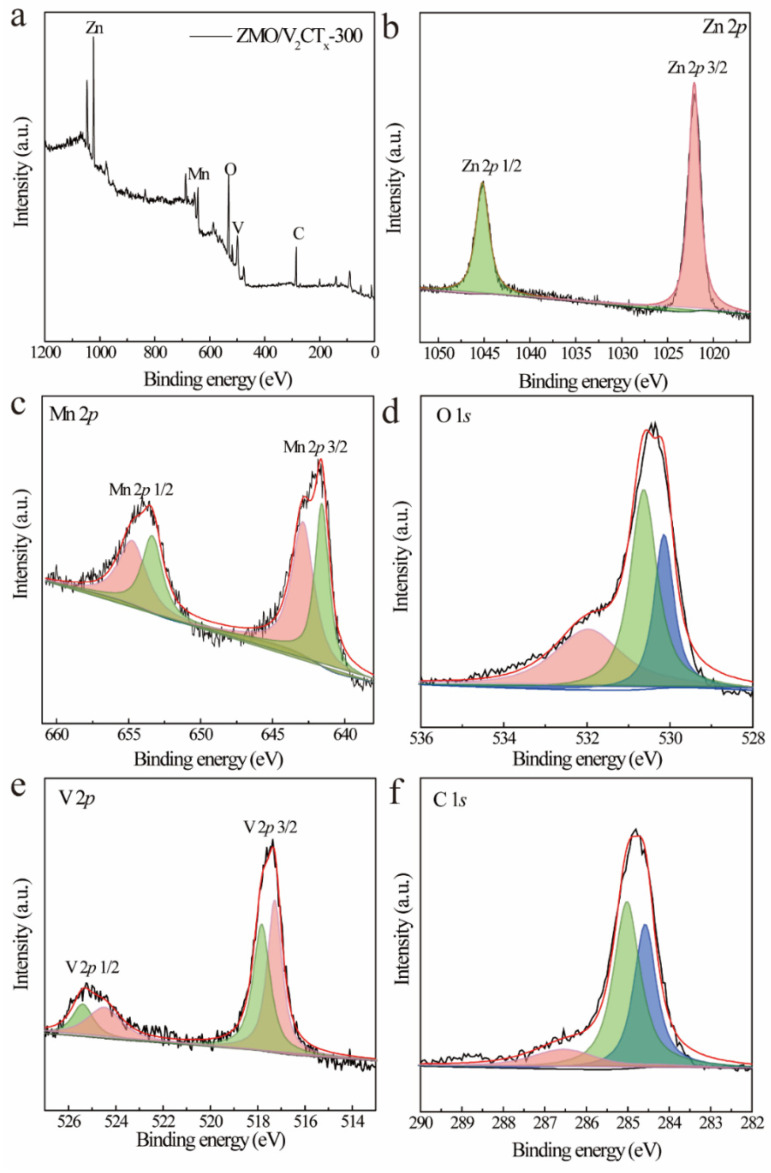
XPS spectra of ZMO/V_2_CT_x_−300: XPS profiles (**a**); Zn 2*p* spectra (**b**); Mn 2*p* spectra (**c**); O 1*s* spectra (**d**); V 2*p* spectra (**e**); C 1*s* spectra (**f**). Black is the experimental curve, and red is the fitting line.

The change in the valence state of ZMO/V_2_CT_x_-300 was investigated using XPS. The obtained spectra from the investigation (Figure 4a) indicate the presence of elements, including zinc (Zn), manganese (Mn), oxygen (O), vanadium (V), and carbon (C).

In Figure 4b, the observed peaks corresponding to energy levels of 1022.1 and 1045.05 eV can be assigned to the Zn 2*p* 3/2 and Zn 2*p* 1/2 orbitals, respectively. Similarly, in Figure 4c, the Mn 2*p* spectra exhibit two separate peaks at energies around 641.65 and 653.95 eV, suggesting the presence of distinct states corresponding to Mn 2*p* 3/2 and Mn 2*p* 1/2. In particular, it is worth noting that the peak corresponding to Mn^3+^ can be further resolved into four distinct peaks at approximate binding energies of around 641.6 eV (653.4 eV) and around binding energies of approximately 642.95 eV (654.8 5eV), which are attributed to different oxidation states: Mn^3+^ and Mn^4+^. The analysis of the O1s spectrum (Figure 4d) exhibited three separate peaks at binding energies of 530.15, 530.65, and 531.95 eV, respectively, suggesting the existence of diverse oxygen components on the surface. These peaks correspond to Mn-bonded lattice oxygen (OL), oxygen vacancy sites (Ov), and surface-absorbed hydroxyl groups (-OH). The oxygen vacancies act as adsorption sites for various oxygen species like O^−^, O^2−^, and Mn-OH. The presence of adsorbed surface oxygen components is responsible for the observed peak at 530.65 eV, which affects the oxygen vacancy state of the active substance. The V 2*p* spectrum depicted in Figure 4e displays two distinct peaks at approximately 517.45 and 525.25 eV, representing the binding energies of V 2*p* 3/2 and V 2*p* 1/2, respectively. Specifically, the V 2*p* spectrum can be accurately modeled with four peaks: V^4+^ at around 525.4 and 517.85 eV, as well as the coexisting V^5+^ at approximately 524.5 and 517.25 eV. Furthermore, the C1s spectra (Figure 4f) were fitted using a three-peak model with assignments made for C=O, C-O, and C-C at energies of about 286.55, 285, and 284.6 eV, respectively. The XPS results confirm the successful synthesis of the ZMO/V_2_CT_x_-300 composite.

### 3.2. Electrochemical Performance

**Figure 5:**
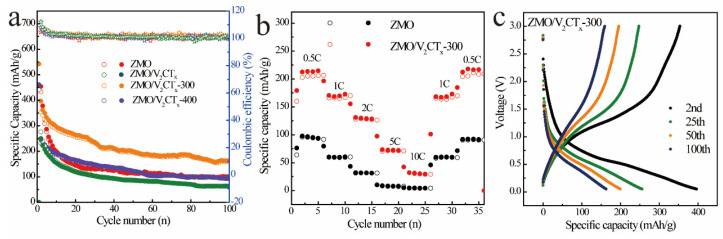
Cycling performance of ZMO and ZnMn_2_O_4_/V_2_CT_x_ electrodes (**a**); rate performance of ZMO and ZMO/V_2_CT_x_-300 electrodes (1C = 100 mAh/g) (**b**); GCD profiles of ZMO/V_2_CT_x_-300 electrode (**c**).

The cyclic behavior of the ZMO and ZnMn_2_O_4_/V_2_CT_x_ electrodes is depicted in Figure 5a. The ZMO/V_2_CT_x_-300 electrode demonstrates superior cycling performance compared to ZMO, exhibiting a remarkable reversible capacity of 163 mAh g^−1^ after undergoing 100 cycles. In contrast, the capacity achieved by the ZMO electrode is lower at 99.5 mAh g^−1^. It is important to mention that the Coulombic efficiency of the ZMO/V_2_CT_x_-300 electrode remains consistently close to 100% throughout continuous cycling, providing additional evidence supporting the electrochemical stability of this composite. The distinct advantages can be ascribed to the composite nature of the material and its calcination process, which enhance ion diffusion pathways and afford a greater abundance of active sites. Figure 5b illustrates the average discharge capacities at various current rates, where the values of 212.9, 169.4, 129.1, 72.4, and 29.7 mAh g^−1^ correspond to current rates of 0.5, 1, 2, 5, and 10 C, respectively. Furthermore, after undergoing cycling at different current densities, electrodes demonstrated discharge capacities around approximately 167.99 and 216.3 mAh g^−1^ when operated at rates of 1 and 0.5 C, respectively. The electrochemical charge–discharge curve shown in Figure 5c depicts the performance of ZMO/V_2_CT_x_-300 electrodes when discharged and charged at a rate of 1 C. In the second cycle, these electrodes exhibited a discharge capacity of 396.7 mAh g^−1^, which remained consistent at a specific capacity of 163 mAh g^−1^, even after undergoing 100 cycles. Additionally, it is worth noting that the charge–discharge curves have maintained their shape since the second cycle, indicating remarkable periodic electrochemical properties of the material.

**Figure 6:**
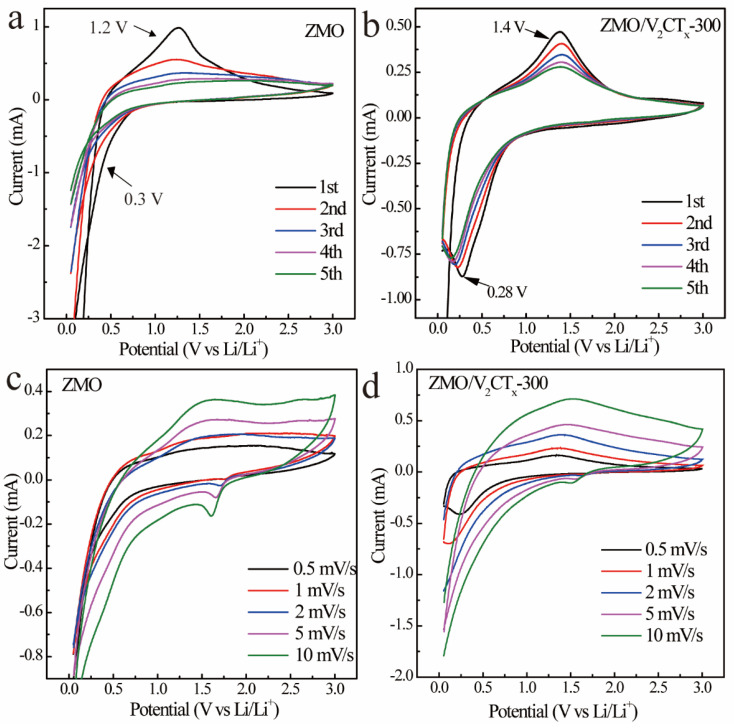
CV curves of ZMO and ZMO/V_2_CT_x_-300 electrodes at 1 mV s^−1^ (**a**,**b**); CV curves of ZMO and ZMO/V_2_CT_x_-300 electrodes at different scan rates (**c**,**d**).

The CV analysis was conducted on the ZMO and ZMO/V_2_CT_x_-300 cells to investigate the underlying reaction mechanism and chemical kinetics. Figure 6a,b illustrate the CV curves of the ZMO and ZMO/V_2_CT_x_-300 electrodes, respectively, using a scanning rate of 1 mV s^−1^. It can be observed that the maximum point observed at around 1.2 V in the cyclotron voltammetry curve of ZMO corresponds to the conversion of Mn^3+^ into Mn^2+^. Similarly, the peak at approximately 0.3 V corresponds to the transformation of both Mn^2+^ and Zn^2+^ ions into elemental forms of Mn and Zn, respectively, resulting in the subsequent creation of the Li-Zn alloy at this specific potential. This can be represented by Equation [20]:

ZnMn_2_O_4_ + 8Li^+^ + 8e^−^ ↔ Zn + 2Mn + 4Li_2_O, (1)

Zn + *x*Li^+^ + *x*e^−^ ↔ Li*_x_*Zn (0 < *x*< 1). (2)

The subsequent similarity of the curves demonstrates the robustness of the ZMO electrochemical properties. In comparison to Figure 6a,b exhibits certain alterations, such as a shift in its oxidation peak from 1.2 to 1.4 V, which aligns more closely with the oxidation peak position observed for V_2_CT_x_. The corresponding reaction equations for this transformation are provided below [21]:

V_2_C/V_2_CY_2_ + *x*Li^+^ + *x*e^−^ ↔ V_2_CLi*_x_*/V_2_CY_2_Li*_x_* (Y = F/OH). (3)

The chemical kinetics of the cell were investigated using cyclovoltammetry at various scan rates (0.5, 1, 2, 5, and 10 mV s^−1^), as depicted in Figure 6c,d. It was noted in the CV spectrum that a higher scanning rate led to a displacement of the oxidation peak towards higher potential, a movement of the reduction peak towards lower potential, and an enlargement of the current area due to Li^+^ polarization during continuous insertion and extraction processes.

## 4. Conclusions

In summary, ZnMn_2_O_4_/V_2_CT_x_ composites were synthesized through high-temperature calcination. The results demonstrate that the structure and morphology of the ZnMn_2_O_4_/V_2_CT_x_ material undergo alterations upon different temperature treatments. At 400 °C, the Mn_2_V_2_O_7_ phase emerges, leading to a transformation in the morphology of the ZnMn_2_O_4_/V_2_CT material from rod-like bonds to spherical structures and subsequently scaffold-like structures. Despite undergoing 100 cycles at a nominal specific capacity of 100 mAh g^−1^, the ZMO/V_2_CT_x_-300 electrode exhibits an enduring specific discharge capacity of 163 mAh g^−1^. This signifies an estimated surge of around 64% in contrast to the discharge capability (99.5 mAh g^−1^) witnessed in the electrode composed solely of ZnMn_2_O_4_. These findings suggest that recombination and calcination processes effectively enhance both ionic transport capacity and conductivity within ZnMn_2_O_4_ materials. This research offers valuable perspectives on the effective utilization of AB_2_O_4_-based materials and MXene materials as electrodes with exceptional performance.

## Data Availability

The raw/processed data required to reproduce these findings cannot be shared at this time as the data also forms part of an ongoing study.
